# Preservation of Phenols, Antioxidant Activity, and Cyclic Adenosine Monophosphate in Jujube (*Ziziphus jujuba* Mill.) Fruits with Different Drying Methods

**DOI:** 10.3390/plants12091804

**Published:** 2023-04-28

**Authors:** Govinda Sapkota, Efren Delgado, Dawn VanLeeuwen, F. Omar Holguin, Nancy Flores, Shengrui Yao

**Affiliations:** 1 Department of Plant and Environmental Sciences, New Mexico State University, Las Cruces, NM 88003, USA; 2 Center of Excellence in Sustainable Food and Agricultural Systems, New Mexico State University, Las Cruces, NM 88003, USA; 3 Department of Economics, Applied Statistics, and International Business, New Mexico State University, Las Cruces, NM 88003, USA; 4 Department of Extension Family and Consumer Sciences, New Mexico State University, Las Cruces, NM 88003, USA; 5 Sustainable Agriculture Science Center, New Mexico State University, Alcalde, NM 87511, USA

**Keywords:** antioxidant activity, bioactive compounds, convective oven drying, cyclic adenosine monophosphate, freeze drying, proanthocyanidins, sun drying, total phenolic content, vitamin C

## Abstract

Jujube, commonly known as the Chinese date, is a nutritious fruit with medicinal importance. Fresh jujube fruits have a shelf life of about ten days in ambient conditions that can be extended by drying. However, nutrition preservation varies with the drying method and parameters selected. We studied total phenolic content (TPC), proanthocyanidins (PA), vitamin C, cyclic adenosine monophosphate (cAMP), and antioxidant activities in jujube fruits dried with freeze-drying (FD), convective oven drying (OD) at 50 °C, 60 °C, and 75 °C, and sun drying (SD) with FD as a control. The cultivars used for this study were ‘Capri’ and ‘Xiang’ from Las Cruces in 2019, and ‘Sugarcane’, ‘Lang’, and ‘Sherwood’ from Las Cruces and Los Lunas, New Mexico, in 2020. Freeze-drying had the highest of all nutrient components tested, the best estimates of mature jujube fruits’ nutrient contents. Compared with FD, the majority of PA (96–99%) and vitamin C (90–93%) was lost during SD or OD processes. The retention rates of antioxidant activities: DPPH and FRAP were higher in OD at 50/60 °C than SD. SD retained a higher cAMP level than OD at 50/60 °C in both years. The increase in oven drying temperature from 60 °C to 75 °C significantly decreased TPC, PA, antioxidant activities, and cAMP.

## 1. Introduction

Jujube (*Ziziphus jujuba* Mill.) originated in China over 4000 years ago and belongs to the Rhamnaceae family. Jujube fruits are a good source of vitamin C (Vc), polyphenols, cyclic adenosine monophosphate (cAMP), polysaccharides, and triterpenic acids [1,2,3]. It is one of the essential medicinal herbs in Chinese traditional medicine for analeptic, palliative, and antibechic purposes [4]. Jujube is commercially available primarily in fresh and dried forms [1,4]. Jujube fruits cannot be held fresh for more than ten days at ambient conditions because of rapid postharvest ripening [5,6]. The faster deterioration of fresh jujube fruits within a few days after harvest reduces their commercial value, and preserving their commercial value requires drying to extend shelf life. In addition to extending shelf life by preventing microbial growth and reproduction, drying also reduces volume and weight, assisting transportation and storage. Technological advancements have made it possible to preserve fresh jujubes in a fresh state for a period ranging from two-to-four months using controlled atmospheric storage or controlled freezing-point storage [7]. However, the year-round supply of jujube fruits still relies on dried forms. Dried fruits accounted for 95% of the total jujube production in China in the past. Dried jujubes (whole and sliced), as well as jujube powder, have a wide range of potential uses such as medicinal use, culinary use, baking use, herbal tea, snacking, and as a natural sweetener and flavor enhancer [8].

Selecting the proper drying method/parameters is essential to preserve the nutrients and sensory attributes of jujube fruits because the degradation of different bioactive compounds varies with the drying method/parameter and the type of compound [5,9,10]. Innovative drying techniques such as freeze drying (FD), vacuum drying (VD), short-and medium-wave infrared radiation drying (SMIR), microwave drying (MD), microwave vacuum freeze-drying (MVFD), microwave vacuum puffing (MVP), and vacuum freeze-drying (VFD) have been studied with jujube fruits [8]. However, convective oven drying and sun drying are more feasible for small jujube growers and processors. Despite the low cost, not all locations are suitable for sun drying, and its effectiveness depends on specific climatic conditions, such as a daily maximum temperature of 30 °C or higher and humidity levels below 60% [11].

Cultivar and growing location have been demonstrated to impact the nutrient profile of jujubes, which could cause variations in the quality of dried jujubes during thermal processing. This study involved jujube fruits of different cultivars grown in different locations with distinct climatic conditions in New Mexico, U.S. As the jujube is a less studied minor fruit crop in the U.S., drying studies with the U.S.-grown cultivars will help small growers and processors select appropriate drying methods and educate consumers about the nutritional facts of jujubes. More information is also needed on the effect of drying methods, particularly for cyclic nucleotides (cAMP) found in jujubes. In addition, the increased health consciousness of consumers also demands dried jujubes and their products with optimum nutritional values. Therefore, this study evaluated health-promoting compounds such as total phenolic content, proanthocyanidins, vitamin C, cyclic adenosine monophosphate, and antioxidant activities in jujube fruits with freeze-drying (control), convective oven drying (OD) at 50 °C, 60 °C, and 75 °C, and sun drying (SD). This study’s findings may guide growers and processors to choose the suitable drying method for preserving nutrients in jujubes based on available resources, marketing options, and consumer demands and preferences.

## 2. Results

### 2.1. Color Preservation

**Figure 1:**
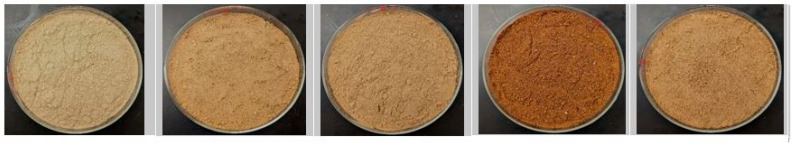
Dried jujube fruits’ powder of cultivar Xiang from left to right: freeze drying, convective oven at 50 °C, 60 °C, 75 °C, and sun drying.

Color is an important sensory quality in products. In our results, the main effects of the drying method were significant for L*, a*, and ΔE, while the main effects of the drying method and cultivar were significant for b*. The L* value was the highest for freeze drying, followed by sun drying, oven drying at 60 °C, and oven drying at 75 °C, respectively (Table 1). A significant decrease in L* values in oven-dried and sun-dried samples compared to freeze-dried samples indicated a decrease in lightness and the color of dried powder becoming dark. An increase in redness and a decrease in greenness were indicated by a*. Oven-dried powder at 75 °C had the highest redness compared to other samples. Freeze-dried samples had a* value of −2.97, indicating the greenish color of freeze-dried powder. There were no significant differences in a* values between sun-dried samples and samples oven-dried at 60 °C. The value of b* represented yellowness. Yellowness was highest in samples oven dried at 60 °C, while freeze-dried samples had the lowest b* values (Table 1). ΔE reflected the total color differences between freeze-dried and dried samples using other methods. ΔE value was the highest for samples oven-dried at 75 °C, followed by oven dried at 60 °C and sun-dried samples, respectively (Table 1). Yellowness was higher in ‘Xiang’ with b* 28.17 (±0.71) than in ‘Capri’ with the value 25.77 (±0.71). Figure 1 shows jujube powders dried using freeze drying, oven drying at 50 °C, 60 °C, 75 °C, and sun drying. Pictures of jujube fruits (whole) dried with different methods are shown in Figures S3 and S4.

### 2.2. Total Phenolic Content (TPC)

In 2019, TPCs were highest in freeze-dried samples (Control), followed by oven-dried samples at 60 °C; sun-dried samples had lower TPCs than freeze-dried and oven-dried samples at 60 °C, while samples at 75 °C had the lowest TPCs. Samples of freeze-dried, oven-dried at 60 °C, sun-dried, and oven-dried at 75 °C had 12.75, 11.16, 7.74, and 6.64 mg GAE/g DW of TPC estimates, respectively (Table 2). Compared to freeze drying, SD and OD at 60 °C and 75 °C retained 60.8%, 87.5%, and 52.1% of TPCs, respectively.

In 2020, the interaction effect of the cultivar × location × drying method was significant for TPCs. The simple effect of the cultivar within each location and drying method is shown in Figure S1. Averaged across cultivars and locations as well as within each combination of cultivar and location, the TPC estimates were higher for freeze-dried samples than for other drying methods (Table 3). The TPC values in freeze-dried samples, oven-dried samples at 50 °C, and sun-dried samples were 11.62, 6.78, and 6.57 mg GAE/g DW, respectively (Table 3). Compared to freeze drying, SD and OD at 50 °C retained 58.3% and 56.5% of TPC (Table 3).

There were no differences in the TPC retention for OD at 50 °C and SD. Among freeze-dried samples, ‘Sugarcane’ had higher TPCs, followed by ‘Lang’ and ‘Sherwood’ with values of 13.12, 11.55, and 10.19 mg GAE/g DW, respectively (Figure S1). There were no significant differences in the TPCs among the three cultivars when oven-dried at 50 °C (Figure S1). However, sun-dried ‘Sugarcane’ had the highest TPCs, and ‘Sherwood’ had the lowest (Figure S1).

### 2.3. Proanthocyanidins (PA)

In 2019, only the main effect of drying method was significant for PA. Overall, PA was the highest for freeze drying, followed by oven drying at 60 °C, sun drying, and oven drying at 75 °C, respectively (Table 2). Compared to FD, 96 to 97.5% of PA was lost during the drying processes for OD at 60 °C and SD. An increase in oven drying temperature from 60 °C to 75 °C reduced the PA from 4% to 2% of FD treatment. The interaction effect of cultivar × location × drying method was significant for the PA in 2020. Compared to the FD, 98.3% of the PA was lost during the OD at 50 °C and SD treatments (Table 3). The simple effect of cultivar and drying method within each location is shown in Table S1.

### 2.4. Antioxidant Activity (DPPH)

**Figure 2:**
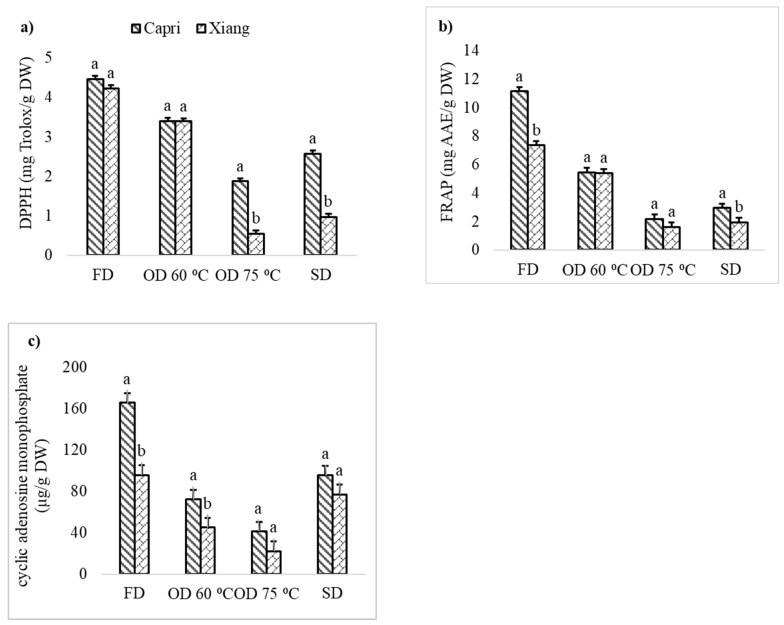
(**a**) Antioxidant activity with 2,2-diphenylpicrylhydrazyl (DPPH), (**b**) Ferric reducing antioxidant potential (FRAP), and (**c**) cyclic adenosine monophosphate (cAMP) in ‘Capri’ and ‘Xiang’ from Las Cruces New Mexico, U.S. in 2019 with different drying methods: FD-freeze drying, OD 60 °C -convective oven drying at 60 °C, OD 75 °C -convective oven drying at 75 °C, SD-sun drying. Bars that share the same letter in each drying method do not differ (*p* ≥ 0.05). Antioxidant activity with 2,2-diphenylpicrylhydrazyl (DPPH), ferric reducing antioxidant potential (FRAP), and cyclic adenosine monophosphate (cAMP) are expressed as mg Trolox/g, milligrams of ascorbic acid equivalent per gram, and microgram per gram, respectively, on a dry-weight basis.

Compared to freeze drying, 27.8% to 78.2% of 2,2-diphenylpicrylhydrazyl (DPPH) values were retained with the SD or OD methods (Table 2 and Table 3). In 2019, overall, the DPPH values were the highest in freeze-dried samples, followed by oven-dried samples at 60 °C, sun-dried samples, and samples oven-dried at 75 °C, respectively (Table 2). As compared to the FD, OD at 60 °C retained higher DPPH activity than the SD and OD at 75 °C, with DPPH retention rates of 78.2%, 40.7%, and 27.8%, respectively. DPPH did not differ between cultivars for FD or OD at 60 °C (Figure 2a). However, ‘Capri’ samples oven dried at 75 °C had a higher DPPH value of 1.87 mg Trolox/g DW compared to ‘Xiang’ with 0.97 mg Trolox/g DW. Sun-dried ‘Capri’ samples had a higher inhibition of DPPH radicals with a value of 2.57 mg Trolox/g DW than the 0.97 mg Trolox/g DW in ‘Xiang’ (Figure 2a). In 2020, the interaction effect of cultivar × location × drying method was significant for DPPH. The simple effect of cultivars within each location and drying method is shown in Figure S2. In 2020, the DPPH values were higher in freeze-dried samples, followed by the oven-dried samples at 50 °C and sun-dried samples, with 3.97, 2.31, and 2.11 mg Trolox/g DW, respectively (Table 3). In each drying method studied in 2020, ‘Sherwood’ had the lowest DPPH, and there were no significant differences between ‘Lang’ and ‘Sugarcane’ (Figure S2).

### 2.5. Ferric Reducing Antioxidant Potential (FRAP)

In 2019, the overall FRAPs were higher in the freeze-dried samples, followed by the oven dried at 60 °C, sun-dried samples, and oven-dried samples at 75 °C, respectively (Table 2). Compared to freeze drying, the SD, OD at 60 °C, and OD at 75 °C retained 26.5%, 58.6%, and 20.6% of FRAP, respectively. Freeze-dried samples of ‘Capri’ had higher FRAPs than ‘Xiang’, with estimates of 11.14 and 7.34 mg ascorbic acid equivalent/g on a dry-weight basis (mg AAE/g DW) (Figure 2b). However, there were no significant differences in FRAPs between the ‘Capri’ and ‘Xiang’ oven dried at 60 °C and 75 °C. The sun-dried ‘Capri’ had a higher FRAP of 2.95 mg AAE/g DW than the ‘Xiang’ with 1.94 mg AAE/g DW.

In 2020, the interaction effect of cultivar × location × drying method was significant for FRAP. The simple effect of cultivar and drying method within each location is shown in Table S2. In 2020, freeze-dried samples had a higher FRAP value of 9.36 mg AAE/g DW, while there were no significant differences in FRAPs between samples oven dried at 50 °C and sun-dried samples (Table 3). Compared to freeze drying, SD and OD at 50 °C retained 21.6 to 23.3 % of FRAP. Among cultivars, Sugarcane had higher FRAPs, followed by Lang and Sherwood in freeze-dried samples (Table S2). FRAP estimates for freeze-dried ‘Sugarcane’, ‘Lang’, and ‘Sherwood’ were 11.26 (±0.25) mg AAE/g DW, 9.31 (±0.25) mg AAE/g DW, and 7.50 (±0.25) mg AAE/g DW, respectively. In each drying method studied, ‘Sherwood’ had the lowest FRAP value (Table S2).

### 2.6. Vitamin C

Only the main effects of cultivar and drying method were significant. Vitamin C was the highest in the freeze-dried samples, followed by the sun-dried samples and oven dried at 60 °C samples (Table 2). Compared to FD, only 7.3–9.6% of vitamin C content was retained in sun-dried and oven-dried samples.

### 2.7. Cyclic Adenosine Monophosphate (cAMP)

In 2019, the highest cAMP content were in the freeze-dried samples, and sun-dried samples had higher cAMP contents than the oven-dried samples (Table 2). Compared to freeze drying, sun-drying and oven drying at 60 °C and 75 °C had cAMP retention rates of 65.9%, 44.9%, and 24.4%, respectively (Table 2). ‘Capri’ had higher cAMP than ‘Xiang’ in freeze drying and oven drying at 60 °C, while there were no significant differences between ‘Capri’ and ‘Xiang’ in the oven drying at 75 °C and sun drying (Figure 2c).

In 2020, the interaction effect of cultivar × location × drying method was significant for cAMP. The simple effect of cultivar and drying method within each location is shown in Table S3. In 2020, cAMP was the highest in freeze-dried samples, followed by sun-dried and oven-dried samples at 50 °C with cAMP retention rates of 74.6% and 69.3%, respectively (Table 3). In each drying method studied in 2020, cAMP was the highest in ‘Lang’ and the lowest in ‘Sugarcane’ (Table S3). Based on the freeze-dried samples, which had the highest cAMP, samples from Las Cruces had higher cAMP than the samples from Los Lunas with the values 146.8 ± 6.85 µg/g DW and 110.2 ± 6.85 µg/g DW, respectively.

## 3. Discussion

### 3.1. Total Phenolic Content and Proathocyanidins

The higher preservation of polyphenols in the freeze-dried samples could be because the freeze-drying prevented the thermal degradation of the compounds and did not allow degradative enzymes to function [9]. In our study, OD and SD retained more than 50% TPCs with OD at 60 °C retaining 87.5% of TPC as compared to FD. However, in 2020, OD at 50 °C retained only 56.5% of TPC, similar to the amount retained by SD. In our samples, the higher oven drying temperature of 75°C retained a fair amount (52%) of TPCs and almost no PA compared to FD. This corresponded with previous research that demonstrated a substantial decrease in flavonols and polymeric proanthocyanidins in jujube samples when the convective oven drying temperature was raised from 50 °C to 70 °C [10]. Another study with pomegranate rinds also demonstrated a significant reduction in total polyphenols with increased convective oven temperatures from 50 °C to 70 °C [12].

The change in the chemical properties of the polyphenols by epimerization, oxidative polymerization, and degradation were responsible for decreased phenolic content [13]. Intermediates of thermal processing, such as carbonyl-containing compounds, interacted directly with the polyphenols through lipid oxidation, the Maillard reaction, and sugar condensation [13]. The drying method/temperature could have a varying degree of impact on different classes of phenolic compounds not considered in our study [5,9,10]. The significant decline in TPCs in sun-dried samples, even though the average daily temperature during drying was low (21.7 °C and 27.9 °C in 2019 and 2020, respectively), could be linked to the presence of polyphenol oxidase (PPO) activity. PPO was an enzyme that catalyzed the oxidation of phenolic compounds, and its level was shown to increase gradually in fresh jujube fruits within six days of being stored at 22 °C [14].

In addition to the drying method and temperature, the sample type, whether whole fruit or slices, could also impact the drying time and nutrient degradation. Our research revealed that oven-dried whole fruits at 75 °C had significantly lower TPCs than sun-dried samples. However, Gao et al. (2012) found that the oven-dried sliced samples (3 mm thickness) at 70 °C for 8 h had significantly higher TPCs than the sun-dried samples [9]. The inconsistencies in the findings could be attributed to variations in cultivars, sample types (whole fruit vs. sliced), drying durations, drying locations, and sun-drying temperatures. Our study suggested that drying jujube fruits at a lower temperature in an oven is a preferable option for preserving polyphenols if freeze drying is not possible. The fact that the sun-dried and oven-dried samples showed almost no preservation of proanthocyanidins implied that they should resort to using freeze drying if a jujube processor intends to target proanthocyanidins and their associated health benefits in dried jujube products.

Our findings suggest the need for exploring the preservation of polyphenols with convective oven drying below 50 °C. To recommend a low-cost drying method for jujube growers and processors, a new sun-drying study could be conducted with blanching treatment before sun drying. These treatments have been shown to increase the retention of polyphenols and antioxidant activity in Indian jujubes (*Ziziphus mauritiana*) [15].

Our study did not cover how the different drying methods affected specific phenolic compounds, the impacts of polyphenol oxidase activity, the formation of compounds such as melanoids with antioxidant properties during high-temperature thermal processing, and the cost-effectiveness of the different drying techniques. All of those would be good future research topics.

### 3.2. Antioxidant Activity (DPPH and FRAP)

Polyphenols, proanthocyanidins, and vitamin C are responsible for antioxidant activity in jujubes [16]. The freeze-dried samples exhibited the greatest antioxidant activity, and the samples dried in an oven at a lower temperature of 50–60 °C showed the second-highest activity. This may be attributed to the fact that drying at higher temperatures of 75 °C and sun drying caused higher degradation of TPC, PA, and vitamin C. Our study showed a strong positive correlation of TPC with antioxidant activities: DPPH (r = 0.878) and FRAP (r = 0.971). Other studies have also reported a positive correlation between TPC and antioxidant activity [16,17]. However, the antioxidant capacities of individual phenolic compounds could vary [18]. Thus, studying drying methods’ effects on individual phenolic compounds could provide better insight into their sensitivity to heat and their antioxidant activities.

### 3.3. Vitamin C

The loss of vitamin C during drying was influenced by moisture, water activity, and temperature during drying [19]. Our samples dried in convective oven temperatures of 60 °C and 75 °C showed significant losses of vitamin C, by 90% specifically, as compared to the freeze-dried samples. This degradation of vitamin C could be attributed to vitamin C’s instability and heat sensitivity [20,21]. The degradation of vitamin C during thermal processing involves complex oxidation and intermolecular rearrangement reactions [21]. Even low temperatures of 30 °C can cause denaturation of vitamin C in different vegetables [20]. Despite a low sun drying temperature of a daily average of 21.7 °C, the degradation of vitamin C by 90% in our samples could be because of exposure to oxygen, prolonged heating in the open air, and exposure to light, which promotes vitamin C degradation [22]. Increasing the oven temperature from 60 °C to 75 °C reduced vitamin C by 24% in our samples. In jujube slices, an increase in air drying temperature from 60 °C to 70 °C led to a reduction in vitamin C by 29%, but there were no significant differences in vitamin C content among jujubes dried at 70 °C, 80 °C, and 90 °C [23]. The vitamin C content of strawberries dried at 70 °C for 9.16 h decreased also by 72% [24]. Therefore, to preserve the health benefits associated with vitamin C in jujube fruits, low-heat methods such as freeze-drying are recommended. Developing value-added products such as low-temperature drinks with freeze-dried jujube powder can provide consumers with the health benefits of vitamin C.

### 3.4. Cyclic Adenosine Monophosphate (cAMP)

The retention of cAMP in the sun-dried samples was significantly higher than in oven-dried samples. This could be because the sun drying temperatures (21.7 °C in 2019 and 27.9 °C in 2020) were lower than the lowest oven drying temperature of 50 °C used in this study. In 2019, retention of cAMP in the oven-dried samples at 75 °C decreased by 45.6% compared to the oven-dried samples at 60 °C. The significant decrease in cAMP with the increase in oven drying temperature was consistent with the findings from Wang et al. (2016), who observed a significant decrease in cAMP as the drying temperature increased from 50 °C to 70 °C in jujube fruits [5]. However, in Wang et al.’s study, sun-dried samples had significantly lower cAMP levels than hot air-dried samples at 50°C, which could be due to differences in the jujube cultivars studied and the climatic conditions of the drying location [5]. Our findings suggested that cAMP is susceptible to thermal degradation, and, therefore, lower temperatures may be preferable for preserving cAMP in jujube fruits.

### 3.5. Drying Methods

Commercially, dried jujube’s moisture content is below 25 % [25,26]. In selecting the appropriate drying method, various factors, such as the cost of drying and the quality of the final product in terms of color, shrinkage, rehydration ability, and nutrient retention, are all crucial. Freeze drying is superior to other drying methods, such as sun drying and oven drying, in preserving nutrients, due to its ability to dehydrate samples through sublimation without causing thermal degradation of the compounds. In freeze drying, several studies showed optimal retention of total phenolic content, flavonoids, antioxidant activities, and vitamin C [5,9,10]. Results from our study also agree with the findings of previous studies. Freeze-dried jujube fruits, however, had inferior natural colors compared to sun-dried and oven-dried jujube fruits (Figures S3 and S4).

While sun drying is a traditional and cost-effective method, it also has limitations. Only fruits with high sugar and acid content are suitable for sun drying. The disadvantages of sun drying include the lack of control over drying parameters, increased microbial contamination, uneven drying, and an inferior final product that can be caramelized or crusted [9]. Humidity below 60% and daily maximum temperatures of 30 °C or higher are ideal for sun drying [11]. Depending on the location, cultivar, and year, jujube fruits reach full-red maturity from early September to early October in New Mexico. Still, by that time, the climate conditions may or may not be suitable for sun-drying. In northern New Mexico, sun-drying jujube fruits can take 10–14 days when the maximum temperatures range from 30.0 to 32.0 °C with plenty of sunshine (early to mid-September). Still, it can take four weeks or longer to dry jujube fruits when the maximum temperatures range from 10.0 to 25.0 °C (late September to mid-October) with precipitation and cloudy days, as shown in Figure S5. We conducted sun drying for our study in 2019 and 2020 in Las Cruces, the southern part of New Mexico, which has a relatively warmer climate than the northern part. Still, due to climatic variations between years, jujube fruits were sun-dried for 21 days in 2019 and 14–15 days in 2020. Before choosing sun drying, growers should consider the location’s weather and climate history.

Although sun drying is a cost-effective method that maintains the natural color of dried fruits (Figures S3 and S4), it decreased the total phenolic content and antioxidant activity in previous studies [9]. Our research confirmed that sun-dried jujube had significantly lower vitamin C content and decreased levels of total phenolic content, proanthocyanidins, and antioxidant activity compared to freeze-dried jujube. However, oven drying can be an alternative in humid areas or for large-scale farmers, with lower temperatures of 50 °C showing better results in terms of nutrient preservation. Our findings suggest that oven drying jujube fruits at the temperature of 50 °C or lower could be an alternative where freeze drying is not possible. However, further studies are required to check the quality of dried jujube fruits at lower oven drying temperatures.

Quality is a multifaceted concept that includes color, shrinkage, nutrient retention, and rehydration capacity. Drying methods should be selected based on the desired end product. It is also important to note that the whole fruits’ drying process may differ from fruit slices or lumps due to differences in heat and mass transfer and mechanical strain behavior [27]. Therefore, before choosing a drying method, growers and processors should carefully consider the marketing options for dried jujube fruits, as they have various uses and values depending on the various attributes of dried jujube fruits.

## 4. Materials and Methods

### 4.1. Sample Collection

In 2019, jujube fruits at full red maturity, fruits with at least 95% peel color red of jujube cultivars Capri (a Sherwood-like cultivar), and Xiang were sampled from three randomly selected trees per cultivar from a replicated jujube cultivar trial at Leyendecker Plant Science Research Center at Las Cruces, New Mexico (lat. 32°12′08.9″ N, long. 106°44′41.4″ W, 1176 m elevation) [28]. In 2020, we sampled fruits from three randomly selected trees of cultivars Sugarcane, Lang, and Sherwood from Leyendecker Plant Science Research Center at Las Cruces and Agricultural Science Center at Los Lunas, NM (lat. 34°46′04.7″ N, long.106°45′45.7″ W, 1478 m elevation). Trees from Las Cruces in 2020 were from the same cultivar trial mentioned for 2019 drying study, while trees from Los Lunas were from a replicated cultivar trial planted in 2012. Samples were transported to the laboratory at New Mexico State University, Las Cruces in ice. Fruits free from diseases and blemishes were freeze-dried (control), sun-dried, and dried in a convective oven at 50 °C, 60 °C, and 75 °C.

### 4.2. Drying Methods

All drying methods used 0.8 to 1 kg of jujube fruits per replication.

#### 4.2.1. Freeze Drying (Control)

Samples were freeze-dried (HarvestRight, North Salt Lake, UT, USA) for about 35 h at −55 °C until the final moisture content of the samples reached below 5%. In 2019, we used whole fruits. However, in 2020, fruits were cut into two halves to accelerate the drying process. Dried samples were ground into a fine powder and stored at −21 °C until further analysis.

#### 4.2.2. Oven Drying

In 2019, samples were dried in a convective oven at 60 °C (Baker’s Pride, Smithville, TN, USA) and 75 °C (Thermo Fisher Scientific Robert-Bosch-Straße 1D—63505 Langenselbold Germany). An increase in oven drying temperature increased the degradation of compounds studied in 2019. Thus, in 2020, samples were oven dried at 50 °C (Baker’s Pride, Allen, TX, USA). Depending on drying temperature, whole fruits were oven dried for 3 to 4 days until the final moisture content reached below 5%. Samples were ground into a fine powder and stored at −21 °C for further analysis.

#### 4.2.3. Sun Drying

Wooden drying frames with several compartments were constructed for drying. Drying frames had stainless steel wire mesh at the base and transparent plastic coverings with small holes at the top to protect fruits from dust and birds. Whole jujube fruits were dried under the sun from 8 am to 6 pm and were kept indoors at night. Drying was stopped after the final moisture content of the fruits reached below 8%. We recorded fruit temperature throughout the drying period using two data loggers. Mean daily temperatures during the drying period were 21.7 °C and 27.9 °C, respectively, in 2019 and 2020. Depending on the cultivar and location, jujube fruits reached full red maturity at different dates. Samples were sun-dried at New Mexico State University, Las Cruces, NM. Fruit maturity that varied with cultivar and location and weather conditions at Las Cruces, NM, accounted for different sun drying periods for different cultivars. In 2019, samples were sun-dried from 3 October to 24 October. In 2020, ‘Sugarcane’ and ‘Lang’ from Las Cruces were dried from 14 September to 28 September. ‘Sherwood’ was dried from 25 September to 11 October. ‘Sugarcane’, ‘Lang’, and ‘Sherwood’ samples from Los Lunas were dried from 16 September to 30 September, 21 September to 5 October, and 10 October to 25 October, respectively. Dried samples were ground into a fine powder and were stored at −21 °C until further analysis.

### 4.3. Chemicals and Reagents

Gallic acid, Folin–Ciocalteau reagent, proanthocyanidin B_2_, sodium carbonate, 2,2-diphenyl-1-picryl-hydrazyl (DPPH), 2,4,6-Tri (2-pyridyl)-s-triazine (TPTZ), ascorbic acid standard, 2,6 dichlorophenolindophenol, and adenosine 3′,5′-cyclic monophosphate were purchased from Sigma Aldrich (Burlington, MA, USA). All other chemicals were of analytical grade purchased from Sigma Aldrich. Trolox (6-Hydroxy-2,5,7,8-tetramethylchroman-2-carboxylic acid) standard was purchased from ThermoFisher Scientific (Waltham, MA, USA).

### 4.4. Extract Preparation for Total Phenolic Content, Proanthocyanidins, and Antioxidant Activity

Dried jujube powder (1.5 g) was mixed with 15 mL of 80% ethanol using a polytron homogenizer (Kinematica CH-6010, Bohemia, NY, USA) for a minute. The mixture was then sonicated in an ultrasonic water bath (ultrasonic bath 15337426, Fischer Scientific, Pittsburgh, PA, USA) at room temperature for 30 min. The total phenolic content (TPC), proanthocyanidins (PA), and antioxidant activity were determined using supernatant filtered through 0.45 µm PVDF syringe filters.

### 4.5. Extract Preparation for Cyclic Adenosine Monophosphate

Dried jujube powder (1.5 g) was mixed with 15 mL of ultrapure deionized water. The mixture was homogenized for one minute using polytron homogenizer (Kinematica CH-6010, Bohemia, NY, USA) and sonicated for 30 min in an ultrasonic water bath (ultrasonic bath 15337426; Fischer Scientific, Pittsburgh, PA, USA) at room temperature. The mixture was centrifuged at 4500 rpm for 10 min at room temperature, and the supernatants filtered through 0.2 µm PVDF syringe filters were used for analysis.

### 4.6. Color Determination

The color of dried jujube fruit powder was measured using a colorimeter (Chroma meter cr 410, Konica Minolta, Tokyo, Japan) calibrated with a standard white ceramic plate before the sample reading. The L* value represented the degree of lightness to darkness, while a* value indicated the degree of redness (+) to greenness (−). The degree of yellowness (+) to blueness (−) was indicated by b* value. The total color difference (ΔE) was determined using the following formula:

(1) $$\Delta E\;=\sqrt{{\left(L-L^{*}\right)}^{2}+{\left(a-a^{*}\right)}^{2}+{\left(b-b^{*}\right)}^{2}}$$

where L, a, and b represent the values of dried jujube powder. L*, a*, and b* represent the values of freeze-dried jujube powder.

### 4.7. Determination of Total Phenolic Content (TPC)

The Folin–Ciocalteau method described by Wang et al. (2011) with modifications was followed to quantify the total phenolic content [29]. The Folin–Ciocalteu reagent (0.5 mL) was added to 0.5 mL of the diluted sample and mixed thoroughly. The mixture was left at room temperature for 5 min in the dark. Then, 1.5 mL of sodium carbonate (20%) solution was added to the mixture and vortexed. The mixture volume was brought to 10 mL with deionized water, vortexed, and incubated for 10 min at 75 °C. The samples’ absorbance readings were taken at 760 nm using a spectrophotometer (ultraviolet-1800, Shimadzu, Kyoto, Japan). Using a gallic acid standard curve, the TPC was expressed as milligrams of gallic acid equivalent per gram of dry weight (mg GAE/g DW).

### 4.8. Determination of Proanthocyaindins (PA) Content

Proanthocyanidins (PA) were determined using the method by Prior et al. (2010) with modifications [30]. First, three mL of Dimethylacetamide (DMAC) reagent was added to 1 mL diluted sample and mixed thoroughly. Then, the mixture was incubated at room temperature. Within 15 to 25 min, the absorbance reading was recorded using a spectrophotometer (ultraviolet-1800, Shimadzu, Kyoto, Japan) set at 640 nm. The proanthocyanidin B_2_ standard curve was used to express PA content as pronathocyanidin B_2_ equivalent per gram on a dry-weight basis (mg proanthocyanidin B_2_/g DW).

### 4.9. Determination of Antioxidant Activity (DPPH)

The method described by Wang et al. (2011) was followed with minor modifications to assess the percent inhibition of 2,2-diphenylpicrylhydrazyl (DPPH) free radicals using jujube extract [29]. Three mL of 0.1 mM ethanolic (80%) DPPH was added to 0.1 mL of jujube extract and vortexed. Then, the mixture was left in the dark at room temperature for 15 min. The absorbance reading at 517 nm was recorded using a spectrophotometer (ultraviolet-1800, Shimadzu, Kyoto, Japan) against an ethanol blank, and the percent inhibition of DPPH was calculated using the equation:

% Inhibition of DPPH radical = [(A_0_ − A_1_)/A_0_)] × 100 (2)

where A_0_ is the absorbance of control (3 mL of DPPH reagent + 100 µL of 80% ethanol), the same as the absorbance of the sample. Inhibition of DPPH radical was expressed as mg Trolox/g on a dry-weight basis using a Trolox standard calibration curve with a concentration range from 15.6 to 500 µg/mL.

### 4.10. Ferric-Reducing Antioxidant Potential (FRAP)

The ferric-reducing antioxidant potential method by Wang et al. (2011) was followed for the assay [29]. FRAP reagent was prepared by mixing 2.5 mL of a 10 mM 2,4,6-Tri (2-pyridyl)-s-triazine (TPTZ) solution in 40 mM HCl with 2.5 mL of 20 mM FeCl_3_.6H_2_O and 25 mL of 0.3 M acetate buffer at pH 3.6. Then, 0.1 mL of the diluted phenolic extract was mixed with 4 mL of FRAP reagent preheated at 37 °C. The mixture was then incubated at 37 °C for 4 min. The absorbance reading at 593 nm against the blank was recorded using a spectrophotometer (ultraviolet-1800, Shimadzu, Kyoto, Japan). The ascorbic acid standard curve was used to quantify and express FRAP values as a milligram ascorbic acid equivalent/g on a dry-weight basis (mg AAE/g DW).

### 4.11. Determination of Vitamin C

The dried jujube powder (1 g) was blended with 20 mL of 2% oxalic acid solution and filtered. Filtered solutions were used to determine the vitamin C content by the visual titration based on the reduction of 2,6-dichlorophenolindophenol described by Kou et al. (2015) with modifications [16]. Three mL of the filtered solution was diluted to 10 mL with 2% oxalic acid and titrated with 0.717 mM/L of 2,6-dichlorophenolindophenol to the endpoint. Ascorbic acid 200 μg/mL was used to calibrate 2,6-dichlorophenolindophenol solution and the vitamin C content was expressed as a milligram per 100 g on a dry-weight basis (mg/100 g DW).

### 4.12. Determination of Cyclic Adenosine Monophosphate (cAMP)

Analysis of cAMP was performed using Waters Acquity high-performance liquid chromatography (HPLC) system (Waters Corporation, Milford, MA, USA) coupled with Quattro Ultima mass spectrometer (Wythenshawe, Manchester, UK). Ten μL samples were passed through 2 × 50 mm, 2.5 μm Synergi^TM^ column (Phenomenex Inc., Torrance, CA, USA) with 0.1% aqueous formic acid (A) and methanol with 0.1% formic acid (B) as the mobile phase. The elution gradient was: 80% A and 20% B (0–1 min), 100% B (1–4 min), and 80% A and 20% B (4–12 min) with the flow rate of 1 mL/min. cAMP content in the sample was quantified using a cAMP standard curve of concentrations 0.78 µg/mL to 100 µg/mL.

### 4.13. Statistical Analysis

Statistical analyses were performed using SAS Version 9.4 (SAS Institute, Cary, NC, USA, 2002–2013) software. The significance level was set at *p* = 0.05. For the 2019 study, we analyzed parameters using a mixed model with fixed effects for cultivar, drying method, and their interactions. As randomly selected fruits from individual trees were assigned to each drying method, we recognized drying method as a repeated or subplot factor. Correlations among drying methods for fruits from the same individual tree were initially modeled using a compound symmetric (CS) covariance structure. In cases where residual analysis suggested non-constant variance, we used heterogenous compound symmetry (CSH) and unstructured covariance matrix (UN), respectively, for proanthocyanidins and vitamin C. For the 2020 data, the analysis was similar but incorporated location as a fixed factor along with cultivar, drying method, and all interactions among these three factors. An initial analysis incorporated a CS covariance structure, but CSH (used for cyclic adenosine monophosphate and ferric reducing antioxidant potential) and UN (for proanthocyanidins) were considered as alternatives because for these variables residual analysis suggested non-constant variance among the drying methods. For all fitted models, we treated drying method as the repeated factor, fitted the model using REML, and computed denominator degrees of freedom using the Kenward-Roger method.

We were primarily interested in the effects of drying method and cultivar, with interest in effects of drying method averaged across locations and cultivars. When an interaction was significant, means separation was applied to the highest order significant interaction. Thus, when the location × cultivar × drying method interaction was significant, the least square means were calculated and means separation letters for the simple effects of drying method and cultivar were determined. The drying methods’ least square means were reported and, when the drying methods’ main effects were significant, means separation was conducted. Means separation was applied to significant cultivar main effect only in the absence of interaction. Correlations were determined from 2020 data.

## 5. Conclusions

Sun drying or low-temperature oven drying could be used to market dried jujubes (whole) with better natural color. Although jujube fruits are known as natural vitamin C pills, drying them in high temperatures in an oven or under the sun can significantly decrease their vitamin C content. Thus, low-heat methods such as freeze-drying are advisable to preserve vitamin C and associated health benefits. Drying jujubes in an oven at temperatures higher than 60 °C is not advisable since it can substantially decrease polyphenols, vitamin C, cAMP, and antioxidant activities compared to freeze-drying. In the future, it may be worthwhile to explore the consequences of convective oven drying temperatures below 50 °C. Sun-dried jujubes can be utilized to create value-added processed products with high potential health benefits associated with cAMP. Further investigation could be performed to analyze the effect of drying temperature and method on the individual phenolic compounds, which are reported to have different sensitivity to heat and could also vary among cultivars. A sun-drying study might be conducted using blanching treatment to explore blanching’s effect on drying rate and nutrient retention. Sun-dried jujubes can be utilized to create value-added processed products with high potential health benefits associated with cAMP. Although freeze-drying was superior in preserving nutrients, consumer preference for freeze-dried products with the studied cultivars is unknown. Therefore, future studies can include rehydration capacity, shrinkage, texture, and consumers’ preferences. In addition, developing different jujube products, such as low-temperature drinks with freeze-dried jujube powder, can offer nutritious choices to consumers.

## Figures and Tables

**Table 1:** Colorimetric values of dried jujube powder of Xiang and Capri, 2019, with different drying methods. Values ± SE are estimates average across cultivars.

Drying Method	L* (Lightness) ^#^	a* (Redness) ^#^	b* (Yellowness) ^#^	ΔE
Freeze drying	82.67 ± 0.97 a	−2.98 ± 0.33 c	21.24 ± 0.61 c	_
Oven drying 60 °C	59.60 ± 0.97 c	7.67 ± 0.33 b	30.35 ± 0.61 a	27.04 ± 1.65 b
Oven drying 75 °C	43.06 ± 0.97 d	11.34 ± 0.33 a	28.02 ± 0.61 b	42.77 ± 1.65 a
Sun drying	63.70 ± 0.97 b	7.47 ± 0.33 b	28.28 ± 0.61 b	22.94 ± 1.65 c

^#^ Within a column, values (± SE) that share the same letter do not differ (*p* ≥ 0.05).

**Table 2:** Total phenolic content (TPC), proanthocyanidins (PA), antioxidant activity with 2,2-diphenylpicrylhydrazyl (DPPH), ferric reducing antioxidant potential (FRAP), vitamin C, and cyclic adenosine monophosphate (cAMP) in jujube fruits of cultivars Capri and Xiang harvested from Las Cruces, New Mexico, U.S. in 2019 with different drying methods. Values ± SE are estimates average across cultivars.

Variable	Freeze Drying	Oven Drying 60 °C	Oven Drying 75 °C	Sun Drying
Total phenolic content (TPC) ^#^ (mg GAE/g, DW)	12.75 ± 0.31 A	11.16 ± 0.31 B	6.64 ± 0.31 D	7.75 ± 0.31 C
Proanthocyanidins (PA) ^#^ (mg PB_2_/g, DW)	2.46 ± 0.20 A	0.10 ± 0.00 B	0.05 ± 0.00 D	0.06 ± 0.00 C
Antioxidant activity (DPPH) ^#,^* (mg Trolox/g, DW)	4.35 ± 0.06 A	3.40 ± 0.06 B	1.21 ± 0.06 D	1.77 ± 0.06 C
Ferric reducing antioxidant potential (FRAP) ^#,^* (mg AAE/g, DW)	9.25 ± 0.15 A	5.42 ± 0.15 B	1.91 ± 0.15 D	2.45 ± 0.15 C
Vitamin C ^#^ (mg/100 g, DW)	1060.12 ± 26.26 A	101.27 ± 7.26 BC	76.90 ± 4.77 C	100.39 ± 4.15 B
Cyclic adenosine monophosphate (cAMP) ^#,^* (µg/g, DW)	130.59 ± 4.57 A	58.60 ± 4.57 C	31.86 ± 4.57 D	86.04 ± 4.57 B

^#^ Within a row, values (± SE) that share the same letter do not differ (*p* ≥ 0.05). * The interaction effect of cultivar × drying method was significant only for DPPH, FRAP, and cAMP. The pattern of change in values of the variables across drying method is similar. Please check Figure 2 for more details.

**Table 3:** The total phenolic content (TPC), proanthocyanidins (PA), antioxidant activity with 2,2-diphenylpicrylhydrazyl (DPPH), ferric reducing antioxidant potential (FRAP), and cyclic adenosine monophosphate (cAMP) in jujube fruits of cultivars Lang, Sherwood, and Sugarcane harvested from Las Cruces and Los Lunas, New Mexico, U.S. in 2020 with different drying methods. Values ± SE are estimates average across locations and cultivars.

Variable	Freeze Drying	Oven Drying 50 °C	Sun Drying
Total phenolic content (TPC) ^#,^* (mg GAE/g, DW)	11.62 ± 0.14 A	6.57 ± 0.14 B	6.78 ± 0.14 B
Proanthocyanidins (PA) ^#,^* (mg PB_2_/g, DW)	4.65 ± 0.11 A	0.06 ± 0.00 B	0.06 ± 0.00 B
Antioxidant activity (DPPH) ^#,^* (mg Trolox/g, DW)	3.97 ± 0.04 A	2.31 ± 0.04 B	2.11 ± 0.04 C
Antioxidant activity (FRAP) ^#,^* (mg AAE/g, DW)	9.36 ± 0.10 A	2.02 ± 0.02 B	2.18 ± 0.03 B
Cyclic adenosine monophosphate (cAMP) ^#,^* (µg/g, DW)	128.56 ± 3.42 A	89.11 ± 1.44 C	95.97 ± 1.92 B

^#^ Within a row, values (± SE) that share the same letter do not differ (*p* ≥ 0.05). * The interaction effect of cultivar × location × drying method was significant for TPC, PA, DPPH, FRAP, and cAMP. Main effects of drying methods shown in the table are because of the similarity in the pattern for drying method within each combination of location and cultivar. For more details, please check Figures S1 and S2 and Tables S1–S3.

## Data Availability

Data will be available upon request.

## Supplementary Materials

The following supporting information can be downloaded at: https://www.mdpi.com/article/10.3390/plants12091804/s1. Figure S1. Total phenolic content (TPC) in different jujube cultivars Lang, Sherwood, and Sugarcane from Las Cruces and Los Lunas, New Mexico, U.S. in 2020 with different drying methods: FD-freeze drying, OD 50 °C-convective oven drying at 50 °C, and SD-sun drying; Figure S2. Antioxidant activity with 2,2-diphenylpicrylhydrazyl (DPPH) in different jujube cultivars Lang, Sherwood, and Sugarcane from Las Cruces and Los Lunas, New Mexico, U.S. in 2020 with different drying methods: FD-freeze drying, OD 50 °C-convective oven drying at 50 °C, and SD-sun drying; Figure S3. Dried jujube fruits of cultivar Capri sampled from Las Cruces, NM in 2019. From left to right: freeze drying, sun drying, and convective oven at 60 °C and 75 °C; Figure S4. Dried jujube fruits of cultivar Sugarcane sampled from Las Cruces, NM in 2020. From left to right: freeze drying, sun drying, and convective oven at 50 °C; Figure S5. Change in fruit weight (%) of ‘Lang’, ‘Sugarcane’, and ‘Sherwood’ during sun drying at Alcalde, New Mexico, U.S. in 2018; Table S1. Proanthocyanidins (PA) in different jujube cultivars Lang, Sherwood, and Sugarcane from Las Cruces and Los Lunas, New Mexico, U.S. in 2020 with different drying methods: FD-freeze drying, OD 50 °C- convective oven drying at 50 °C, and SD-sun drying. Table S2. Ferric reducing antioxidant potential (FRAP) in different jujube cultivars Lang, Sherwood, and Sugarcane from Las Cruces and Los Lunas, New Mexico, U.S. in 2020 with different drying methods: FD-freeze drying, OD 50 °C- convective oven drying at 50 °C, and SD-sun drying; Table S3. cyclic adenosine monophosphate (cAMP) in different jujube cultivars Lang, Sherwood, and Sugarcane from Las Cruces and Los Lunas, New Mexico, U.S. in 2020 with different drying methods: FD-freeze drying, OD 50 °C- convective oven drying at 50 °C, and SD-sun drying.
