# Supplementary material for: Preservation of Phenols, Antioxidant Activity, and Cyclic Adenosine Monophosphate in Jujube (Ziziphus jujuba Mill.) Fruits with Different Drying Methods
Source: Plants (Basel). 2023 Apr 28;12(9):1804. doi: 10.3390/plants12091804 (PMC10181298; doi:10.3390/plants12091804)
Supplement: Supplementary file 1 [file plants-12-01804-s001.zip › plants-2301776-supplementary.pdf]

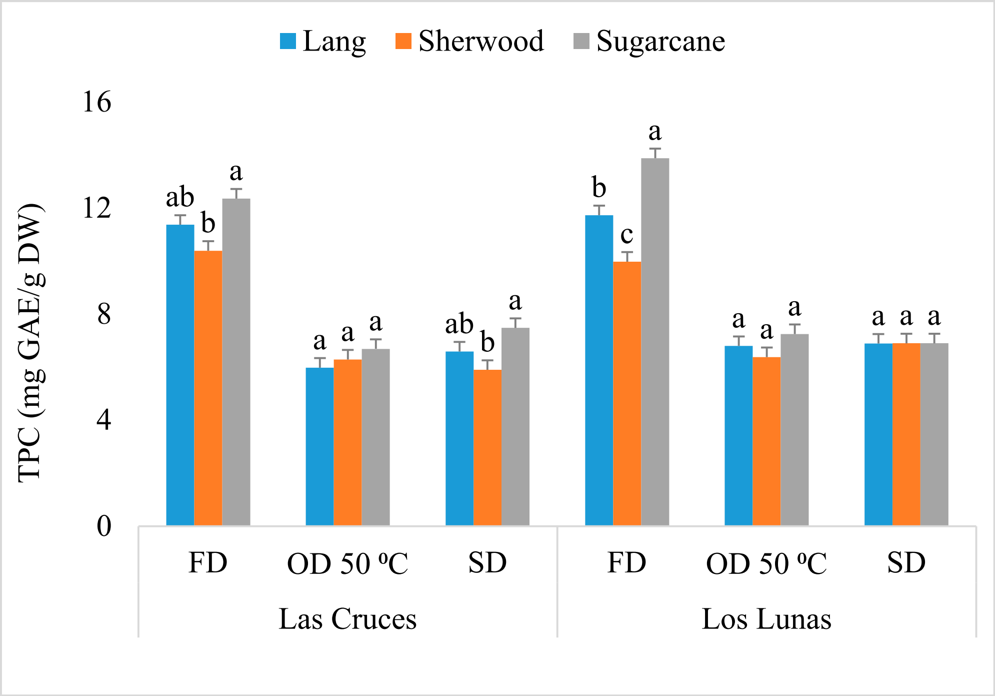

Figure S1. Total phenolic content (TPC) in different jujube cultivars Lang, Sherwood, and Sugarcane from Las Cruces and Los Lunas, New Mexico, U.S. in 2020 with different drying methods: FD-freeze drying, OD 50 °C-convective oven drying at 50 °C, and SD-sun drying. Bars that share same letter in each drying method (location specific) do not differ ($P \geq 0.05$).

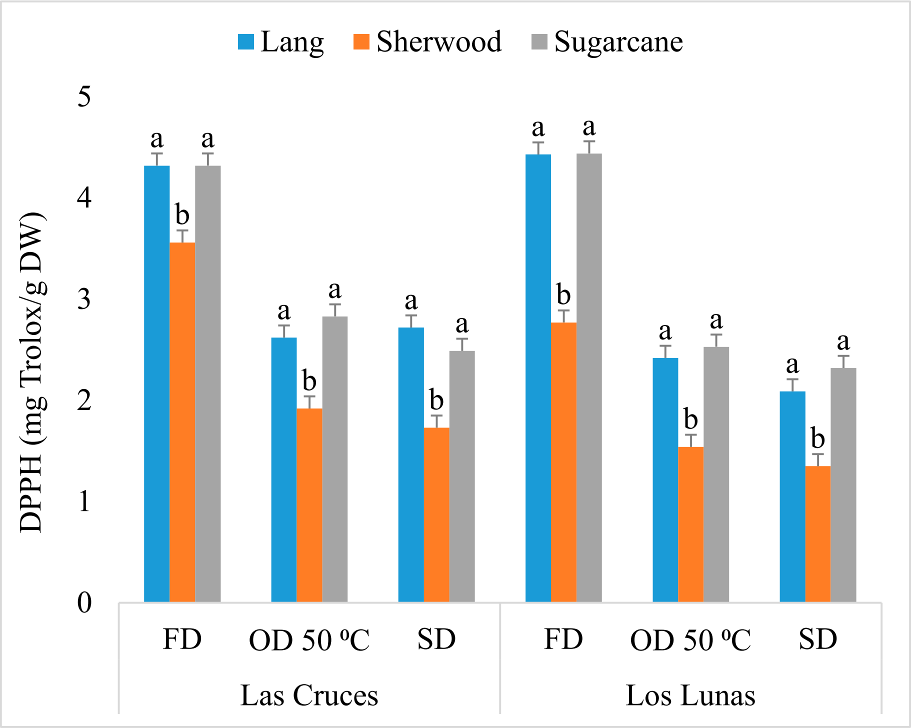

Figure S2. Antioxidant activity with 2,2-diphenylpicrylhydrazyl (DPPH) in different jujube cultivars Lang, Sherwood, and Sugarcane from Las Cruces and Los Lunas, New Mexico, U.S. in 2020 with different drying methods: FD-freeze drying, OD 50 °C-convective oven drying at 50 °C, and SD-sun drying. Bars that share same letter in each drying method (location specific) do not differ ($P \geq 0.05$).

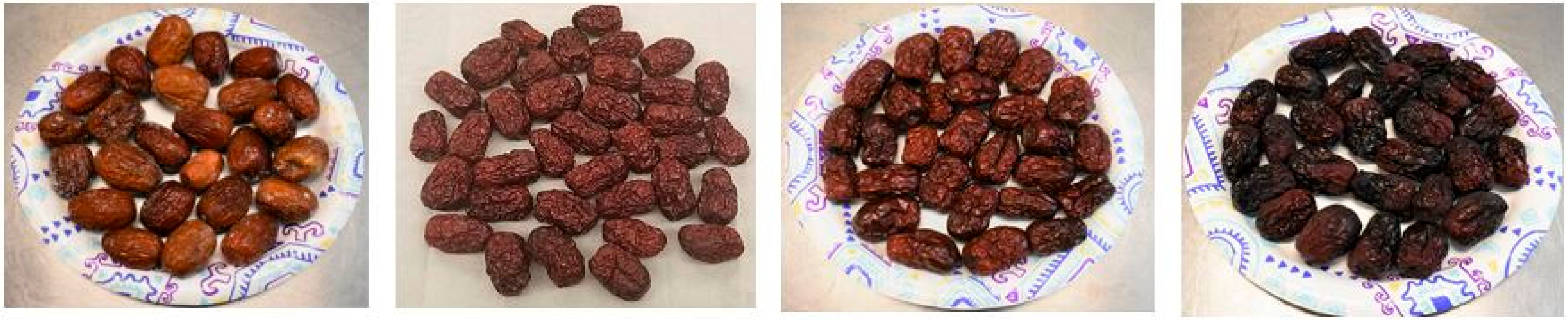

Figure S3. Dried jujube fruits of cultivar Capri sampled from Las Cruces, NM in 2019. From left to right: freeze drying, sun drying, and convective oven at 60 °C and 75 °C.

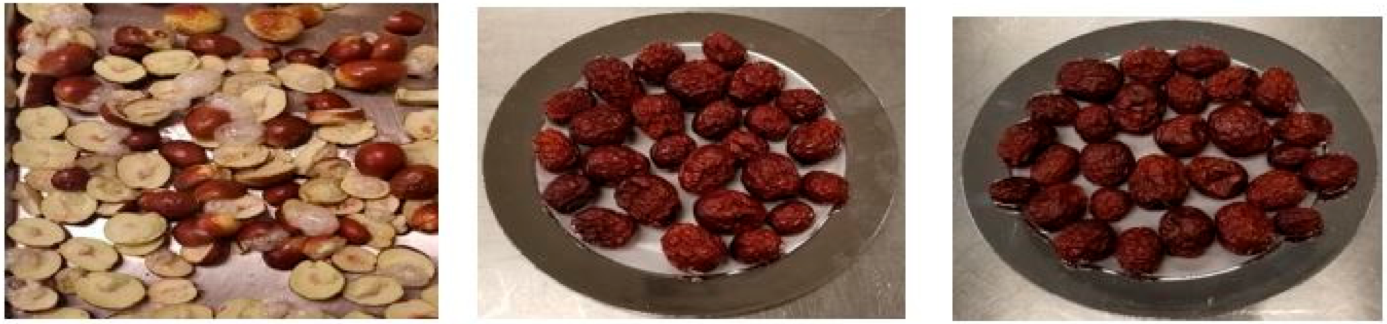

Figure S4. Dried jujube fruits of cultivar Sugarcane sampled from Las Cruces, NM in 2020. From left to right: freeze drying, sun drying, and convective oven at 50 °C.

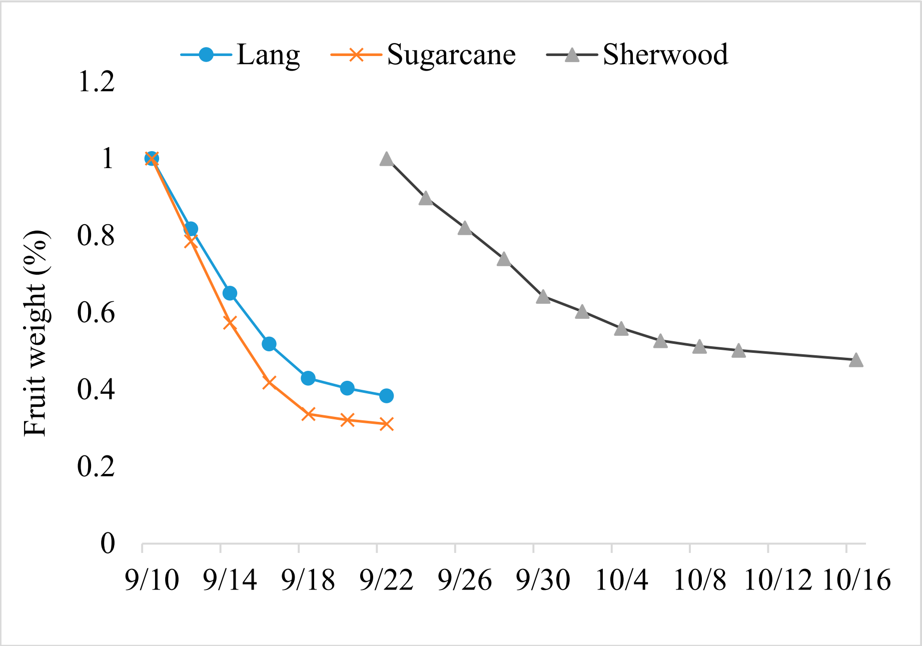

Figure S5. Change in fruit weight (%) of ‘Lang’, ‘Sugarcane’, and ‘Sherwood’ during sun drying at Alcalde, New Mexico, U.S. in 2018.

Table S1. Proanthocyanidins (PA) in different jujube cultivars Lang, Sherwood, and Sugarcane from Las Cruces and Los Lunas, New Mexico, U.S. in 2020 with different drying methods: FD-freeze drying, OD 50 °C- convective oven drying at 50 °C, and SD-sun drying.

	Las Cruces			Los Lunas		
Cultivar	FD	OD 50 ºC	SD	FD	OD 50 ºC	SD
Lang	3.85 ± 0.38 b A	0.08 ± 0.01 b C	0.10 ± 0.01 a B	2.61 ± 0.38 b A	0.06 ± 0.10 b B	0.05 ± 0.01 b B
Sherwood	8.87 ± 0.38 a A	0.00 ± 0.01 c B	0.00 ± 0.01 b B	7.18 ± 0.38 a A	0.00 ± 0.10 c B	0.00 ± 0.01 c B
Sugarcane	3.13 ± 0.38 b A	0.12 ± 0.01 a B	0.10 ± 0.01 a B	2.28 ± 0.38 b A	0.10 ± 0.10 a B	0.10 ± 0.01 a B

[#] Within a column, values ± SE that share same lowercase letter denotes no significant difference among cultivars ($P \geq 0.05$).

[#] Within a row for each location, values ± SE that share same uppercase letter denotes no significant difference among drying method ($P \geq 0.05$).

Proanthocyanidins (PA) is expressed as milligrams of proanthocyanidin B_2 equivalent per gram on a dry-weight basis.

Table S2. Ferric reducing antioxidant potential (FRAP) in different jujube cultivars Lang, Sherwood, and Sugarcane from Las Cruces and Los Lunas, New Mexico, U.S. in 2020 with different drying methods: FD-freeze drying, OD 50 °C- convective oven drying at 50 °C, and SD-sun drying.

	Las Cruces			Los Lunas		
Cultivar	FD	OD 50 ºC	SD	FD	OD 50 ºC	SD
Lang	9.34 ± 0.35 a A	2.37 ± 0.10 a B	2.33 ± 0.12 a B	9.30 ± 0.35 b A	2.43 ± 0.10 b B	2.29 ± 0.12 a B
Sherwood	8.04 ± 0.35 b A	1.61 ± 0.10 b B	1.68 ± 0.12 b B	6.96 ± 0.35 c A	0.69 ± 0.10 c C	1.69 ± 0.12 b B
Sugarcane	10.08 ± 0.35 a A	2.35 ± 0.10 a C	2.61 ± 0.12 a B	12.45 ± 0.35 a A	2.68 ± 0.10 a B	2.49 ± 0.12 a B

Within a column, values ± SE that share same lowercase letter denotes no significant difference among cultivars ($P \geq 0.05$).

Within a row for each location, values ± SE that share same uppercase letter denotes no significant difference among drying method ($P \geq 0.05$).

Ferric reducing antioxidant potential (FRAP) is expressed as milligrams of ascorbic acid equivalent per gram on a dry-weight basis.

Table S3. cyclic adenosine monophosphate (cAMP) in different jujube cultivars Lang, Sherwood, and Sugarcane from Las Cruces and Los Lunas, New Mexico, U.S. in 2020 with different drying methods: FD-freeze drying, OD 50 ℃- convective oven drying at 50 ℃, and SD-sun drying.

	Las Cruces			Los Lunas		
Cultivar	FD	OD 50 ºC	SD	FD	OD 50 ºC	SD
Lang	208.33 ± 11.8 a A	153.99 ± 5.01 a B	141.98 ± 6.68 a C	149.90 ± 11.88 a A	98.44 ± 5.01 a B	124.47 ± 8.40 a C
Sherwood	134.06 ± 11.8 b A	77.88 ± 5.01 b B	77.54 ± 6.68 b B	125.67 ± 11.88 a A	87.40 ± 5.01 b B	93.64 ± 8.40 b B
Sugarcane	98.20 ± 11.8 c A	70.86 ± 5.01 b B	74.40 ± 6.68 b B	55.21 ± 11.88 b AB	46.11 ± 5.01 c B	63.77 ± 8.40 c A

[#] Within a column, values ± SE that share same lowercase letter denotes no significant difference among cultivars (*P* ≥ 0.05).

[#] Within a row for each location, values ± SE that share same uppercase letter denotes no significant difference among drying method (*P* ≥ 0.05).

cyclic adenosine monophosphate (cAMP) is expressed as microgram per gram on a dry-weight basis.
